# The efficacy data of two household cleaning and disinfecting agents on *Lens culinaris Medik* and *Vicia faba* seed germination

**DOI:** 10.1016/j.dib.2021.106811

**Published:** 2021-01-29

**Authors:** Ahmed Abou Elezz, Talaat Ahmed

**Affiliations:** Environmental Science Center (ESC), Qatar University, P.O. Box 2713, Doha, Qatar

**Keywords:** Sodium hypochlorite, Vinegar, Seed germination, Legume crop seeds, Inhibition

## Abstract

A germination test of *Vicia faba* and *Lens culinaris* seeds under the effect of bleach and vinegar was conducted for seven days, and the observations were recorded daily. The completely randomized design (CRD) was used to examine the germination with three replicates at the lab conditions. Six germination parameters were measured, including germination percentage (GP), germination index (GI), mean germination time (MGT), mean germination rate (MGR), vigour index (VI), plus the fresh weight (FW) and dry weight (DW) of *Vicia faba* and *Lens culinaris* seeds. As a legume crop seeds model, the efficacy of four treatment levels from 0.005% to 0.5% of bleach and vinegar on the germination was tested. A chemical analysis was performed using the ion chromatography (IC) to evaluate the effect of chloride and acetate anions uptake on the seedling germination in addition to other essential nutrients. A significant inhibition in seedling growth was observed with increasing the treatment concentration. The maximum inhibition was recorded for both seeds at 0.5%, followed by 0.1% levels, while a positive effect was represented with the lower concentrations. The chemical analysis of the up taking active ingredients was corroborated the germination outputs.

## Specifications Table

**Table:** 

Subject	Agricultural Sciences, Environmental Chemistry, and Pollution
Specific subject area	Crop seed germination, and chemical pollutants
Type of data	Table, figure, chart
How data were acquired	The germination count of ten sterilized seeds in each Petri dish was performed daily by observation for seven days. The germination parameters were evaluated under the effect of four concentrations (0.005%, 0.05%, and 0.1% 0.5%) of bleach and vinegar. The chemical analysis was performed to measure the uptake levels using ion chromatography (882 Compact IC plus, Metrohm AG- Switzerland). The data were analyzed using SPSS Package 25, Minitab version -17.3.1 and MS Excel 365.
Data format	Raw, analyzed
Parameters for data collection	Germination percentage (GP), germination index (GI), mean germination time (MGT), mean germination rate (MGR), vigour index (VI), plus the fresh and dry weight of *Vicia faba* and *Lens culinaris* seeds were tested at laboratory conditions under the effect of four treatment in addition to the uptake of the chemical pollutants.
Description of data collection	Two legume crop seeds (Lentil and Faba bean) were tested for germination parameters under the effect of diluted treatments of household cleaners in addition to the control.
Data source location	Institution: Environmental Science Center (ESC), Qatar University City: Doha Country: Qatar Latitude and longitude: (25 °48′27.70′′N, 51 °20′47.10′′E)
Data accessibility	Link to the Mendeley Data http://dx.doi.org/10.17632/k7vfsd4692.2

## Value of the Data

• This data was useful to understand the potential impact of some household cleaning agents (bleach and vinegar) on the seed germination of Vicia faba and Lens culinaris seeds.
• The data provided in this article can be used by researchers interested in the potential effects of chemicals pollutants on cultivation.
• Furthermore, the dataset introduced in this article can be used to investigate the possible effect of the chemical pollutants leaked into the environment from household cleaners on crop germination.

## 1 Data Description

**Fig. 1:**
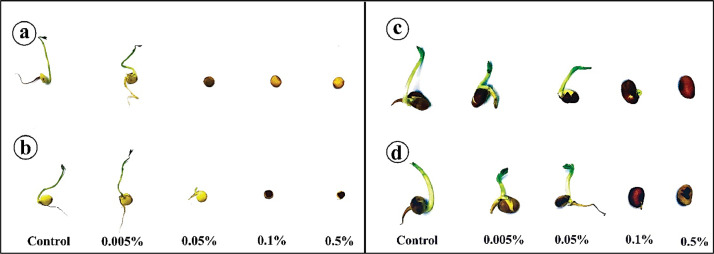
Lentil (*Lens culinaris Medik*) and Faba bean (*Vicia faba*) under the effect of different treatments, (a) lentil with vinegar treatments, (b) lentil with different bleach treatments, (c) Faba bean under different vinegar treatments, and (d) Faba bean under different bleach concentrations.

**Fig. 2:**
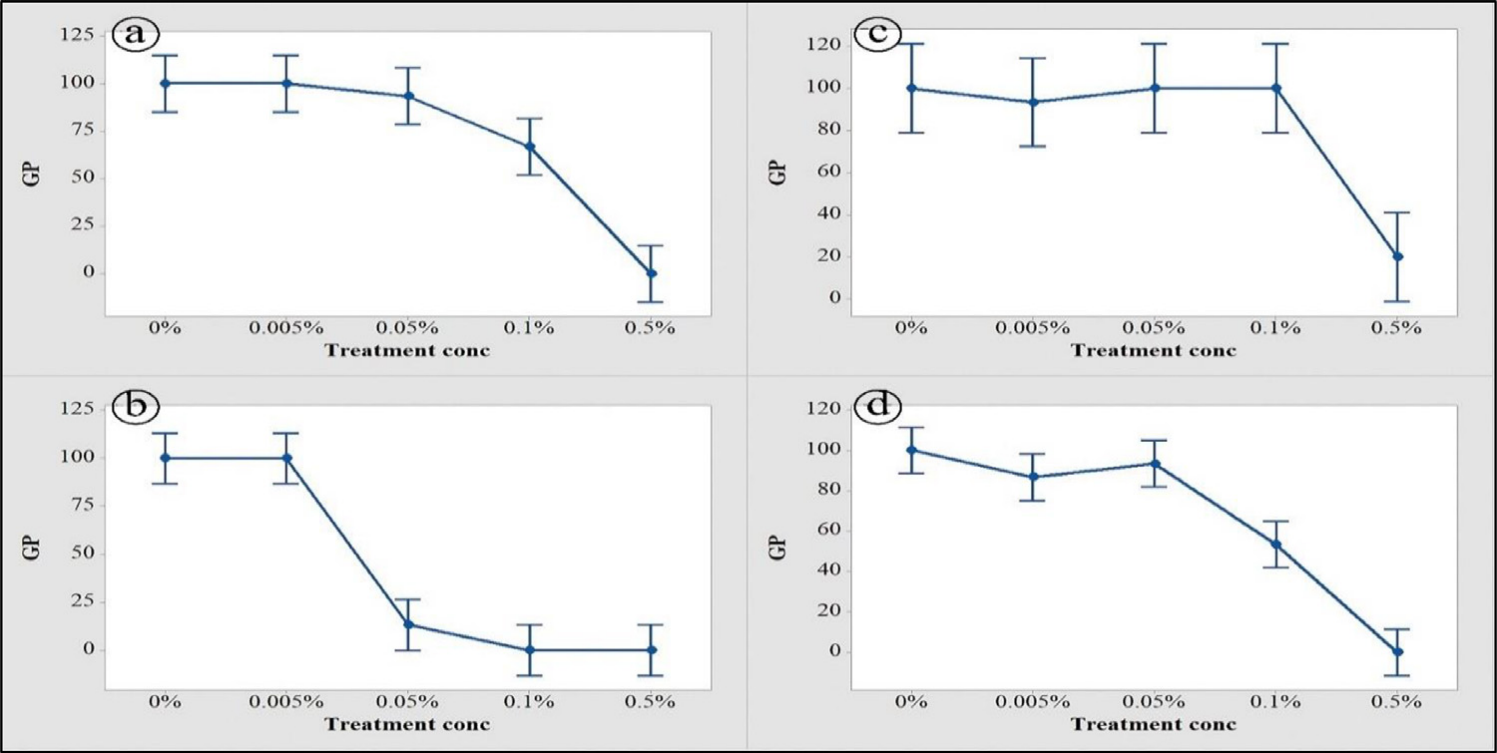
Interval Plot of the mean Germination Percentage (*GP*) of Lentil with bleach treatments (a), Lentil with vinegar treatments (b), Faba bean with bleach treatments (c), and Faba bean with vinegar treatments (d).

**Fig. 3:**
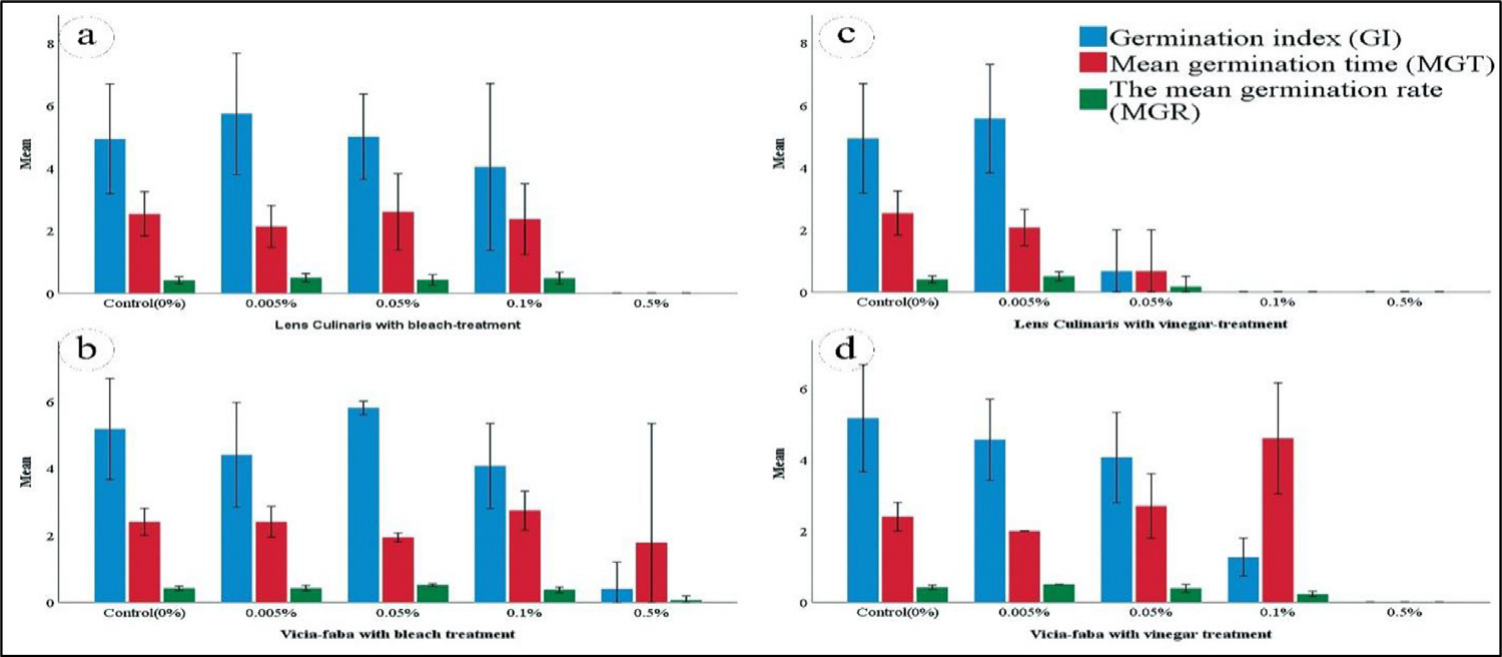
Germination index (*GI*), Mean Germination Time (*MGT*) and Mean Germination Rate (*MGR*) of Lentil with bleach treatments (a), Faba bean with bleach treatments (b), Lentil with vinegar treatments (c), and Faba bean with vinegar treatments (d).

The germination experiment of *Lens culinaris Medik* and *Vicia faba* under different treatment concentrations (0.005%, 0.05%, 0.1%, and 0.5%) of household sanitizers (hypochlorite bleach and white vinegar) was tested for seven days in addition to the control (see Fig. 1 in the supplementary data). Both 0.1 and 0.5% of hypochlorite bleach and white vinegar prevented the seeds of both lentil and faba bean to be germinated alike (Fig. 1). The germination of both seeds under treatments was illustrated in the supplementary data (Figs. 2, 3).

### 1.1 Germination parameters calculations

Six germination parameters were examined during seven days to find out the potential effect of hypochlorite bleach and white vinegar treatments on seed germination of *Vicia faba* and *Lens culinaris* seeds. The observations were recorded daily for both seeds (see the germination parameters test file in the supplementary data). The collected data was used in calculations methodology. Germination percentage Eq. 1) followed Elezz et al. [1], while germination index and mean germination time (Eq. 2, (3) followed Leif Marvin R and Gonzales [2]. The mean germination rate (Eq. 4) was calculated using the formula reported by Al-Ansari & Ksiksi [3]. The coefficient of variation of the time (Eq. 5) was followed by the equation reported by Marli A. Ranal & Denise Garcia De Santana [4]. The vigour index (Eq. 6) was followed by Arya & Gothalwal [5].

(1) $$Germination\;percentage\;=\frac{seeds\;germinated}{total\;seeds\;tested}\times \;100$$

(2) $$Germination\;index\;=\sum_{i=1}^{K}\frac{No.of\;germinated\;seed}{the\;count\;day}$$

Where: *i* =1 day one, k the last day of observation.

(3) $$Mean\;germination\;time=\frac{\sum_{i=1}^{k}n_{i}t_{i}}{\sum_{i=1}^{k}n_{i}}$$

Where: *t_i_* is the time from day one to the last day of observation, *n_i_* is an observed number of germinated seeds every day, and *k* is the last germination time.

(4) $$Mean\;germination\;rate\;=\frac{1}{MGT}$$

(5) $$\mathrm{Coefficient}\;\mathrm{of}\;\mathrm{variation}\;\mathrm{of}\;\mathrm{the}\;\mathrm{time}=\frac{S_{t}}{MGT}\times \;100$$

*S_t_* is a standard deviation of the germination time, and *MGT* is the mean germination time.

(6) $$Vigor\;index=\left({\bar{x}}_{R}\;+{\bar{x}}_{S}\right)\times GP$$

Where: ${\bar{x}}_{R}$ mean root length, ${\bar{x}}_{S}$ mean shoot length (GP) germination per cent.

Germination percentage (GP) was determined by dividing the number of seeds germinated on day seven by the total number of seeds tested (Eq. 1). Germination index (GI) is the summation of the germinated seeds number that counted every day divided by the count day from day one (*i =1*) to the last day of observation (*k*) (Eq. 2). The GI results (in days) were represented in Table 1. Mean germination time (MGT) is defined as the time taken (in days) for seed germination or emerge. Along with Eq. 3, MGT can be calculated, and the obtained results were manifested in Table 1. The mean germination rate (MGR) indicates the speed of seed germination per unit time and is calculated as the inverse of the mean germination time (Eq. 4). The variation coefficient of germination time (CV_t_) was used to measure the germination time homogeneity (Eq. 5). Vigour index (VI) is denoted in Eq. 6 was used to measure the damage accumulations as viability declines [6]. Seedling fresh weight was recorded on day seven, and then samples were allowed to dry in a drying oven (Heratherm, Thermo Fisher Scientific, UK) at 60 °C for 48 h, and the dry weight was calculated [7]. Dry weight is the weight logged after the complete drying of the seedling at 60 °C for 48 h. Table 1 represents the mean fresh weight (FW) and dry weight (DW) in grams of the individual seedling with standard deviation.

**Table 1:** Germination parameters of *Lens culinaris Medik* and *Vicia faba* under the effect of bleach and vinegar treatments (*df=4*) with three replicates.

Crop seed	Sanitizers	Concen-tration	*GP(%)*	*GI (day)*	*MGT (day)*	*MGR (day^−1^)*	*CV_t_ (%)*	*VI*	*FW(g)*	*DW(g)*
*Lens culinaris*	Bleach	0.00%	100.00	6.30	2.00	0.50	47.00	7360.00	0.19	0.06
		0.00%	100.00	3.30	3.20	0.31	25.00	8655.00	0.18	0.05
		0.00%	100.00	5.20	2.40	0.42	45.00	6220.00	0.20	0.06
		0.005%	100.00	6.70	1.80	0.56	44.00	8520.00	0.22	0.06
		0.005%	100.00	6.70	1.80	0.56	44.00	6958.00	0.22	0.06
		0.005%	100.00	3.80	2.80	0.36	28.00	3620.00	0.16	0.05
		0.05%	80.00	3.70	3.80	0.27	65.00	352.00	0.15	0.06
		0.05%	100.00	5.30	2.20	0.45	36.00	1100.00	0.16	0.06
		0.05%	100.00	6.00	1.80	0.56	23.00	540.00	0.16	0.06
		0.10%	80.00	5.00	1.80	0.57	26.00	228.00	0.07	0.06
		0.10%	80.00	5.70	1.80	0.57	51.00	259.20	0.09	0.06
		0.10%	40.00	1.40	3.50	0.29	49.00	56.00	0.07	0.07
		0.50%	0.00	0.00	0.00	0.00	0.00	0.00	0.09	0.06
		0.50%	0.00	0.00	0.00	0.00	0.00	0.00	0.09	0.07
		0.50%	0.00	0.00	0.00	0.00	0.00	0.00	0.07	0.06

	Vinegar	0.00%	100.00	6.30	2.00	0.50	47.00	7360.00	0.19	0.06
		0.00%	100.00	3.30	3.20	0.31	25.00	8655.00	0.18	0.05
		0.00%	100.00	5.20	2.40	0.42	45.00	6220.00	0.20	0.06
		0.005%	100.00	5.70	2.00	0.50	33.00	3600.00	0.25	0.07
		0.005%	100.00	4.00	2.60	0.38	20.00	1880.00	0.20	0.06
		0.005%	100.00	7.00	1.60	0.63	32.00	5580.00	0.16	0.04
		0.05%	40.00	2.00	2.00	0.50	0.00	32.00	0.16	0.06
		0.05%	0.00	0.00	0.00	0.00	0.00	0.00	0.15	0.06
		0.05%	0.00	0.00	0.00	0.00	0.00	0.00	0.15	0.06
		0.10%	0.00	0.00	0.00	0.00	0.00	0.00	0.14	0.06
		0.10%	0.00	0.00	0.00	0.00	0.00	0.00	0.14	0.06
		0.10%	0.00	0.00	0.00	0.00	0.00	0.00	0.14	0.06
		0.50%	0.00	0.00	0.00	0.00	0.00	0.00	0.12	0.06
		0.50%	0.00	0.00	0.00	0.00	0.00	0.00	0.12	0.06
0.50%		0.00	0.00	0.00	0.00	0.00	0.00	0.12	0.06

*Vicia faba*	Bleach	0.00%	100.00	5.30	2.20	0.45	36.00	4810.00	1.90	0.59
		0.00%	100.00	6.40	2.20	0.45	70.00	4745.00	2.06	0.65
		0.00%	100.00	3.80	2.80	0.36	28.00	5160.00	1.88	0.59
		0.005%	100.00	5.70	2.00	0.50	33.00	4640.00	1.74	0.71
		0.005%	100.00	4.50	2.40	0.42	35.00	3120.00	1.74	0.80
		0.005%	80.00	3.00	2.80	0.36	17.00	2624.00	2.12	0.87
		0.05%	100.00	5.70	2.00	0.50	33.00	5640.00	1.64	0.58
		0.05%	100.00	6.00	1.80	0.56	23.00	6400.00	2.13	0.70
		0.05%	100.00	5.70	2.00	0.50	33.00	3420.00	1.89	0.80
		0.10%	100.00	3.70	2.80	0.36	15.00	904.00	1.06	0.65
		0.10%	100.00	5.30	2.20	0.45	36.00	1188.00	1.04	0.61
		0.10%	100.00	3.20	3.20	0.31	13.00	586.00	1.00	0.60
		0.50%	0.00	0.00	0.00	0.00	0.00	0.00	0.77	0.58
		0.50%	0.00	0.00	0.00	0.00	0.00	0.00	0.80	0.60
		0.50%	60.00	1.20	5.33	0.19	19.00	144.00	0.79	0.59

	Vinegar	0.00%	100.00	5.30	2.20	0.45	36.00	4810.00	1.90	0.59
		0.00%	100.00	6.40	2.20	0.45	70.00	4745.00	2.06	0.65
		0.00%	100.00	3.80	2.80	0.36	28.00	5160.00	1.88	0.59
		0.005%	80.00	4.00	2.00	0.50	0.00	2192.00	1.72	0.73
		0.005%	80.00	4.00	2.00	0.50	0.00	2080.00	1.65	0.81
		0.005%	100.00	5.70	2.00	0.50	33.00	3620.00	1.59	0.77
		0.05%	100.00	5.30	2.20	0.45	36.00	1860.00	1.88	0.89
		0.05%	100.00	3.20	3.60	0.28	40.00	2560.00	1.64	0.68
		0.05%	80.00	3.70	2.30	0.44	21.00	1744.00	1.67	0.76
		0.10%	40.00	1.00	4.50	0.22	38.00	40.00	1.23	0.57
		0.10%	60.00	1.80	3.30	0.30	15.00	108.00	1.00	0.49
		0.10%	60.00	1.00	6.00	0.17	0.00	84.00	1.22	0.53
		0.50%	0.00	0.00	0.00	0.00	0.00	0.00	0.90	0.54
		0.50%	0.00	0.00	0.00	0.00	0.00	0.00	0.88	0.49
0.50%		0.00	0.00	0.00	0.00	0.00	0.00	0.93	0.56

**Table 2:** Pearson Correlation coefficients test between *GP* on the one hand and other germination paramitas (*GI, MGT, MGR, CVt,* and *VI*).

		*Lens Culinaris*		*Vicia faba*	
		bleach	vinegar	bleach	vinegar
		
Correlations		Germination percentage (*GP*)			
*GI*	Pearson Correlation	0.910*	0.952*	0.875*	0.907*
	Sig. (2-tailed)	0.000	0.000	0.000	0.000
*MGT*	Pearson Correlation	0.667*	0.933*	0.510*	0.449
	Sig. (2-tailed)	0.007	0.000	0.052	0.093
*MGR*	Pearson Correlation	0.872*	0.905*	0.924*	0.899*
	Sig. (2-tailed)	0.000	0.000	0.000	0.000
*CV_t_*	Pearson Correlation	0.676*	0.917*	0.666*	0.623*
	Sig. (2-tailed)	0.006	0.000	0.007	0.013
*VI*	Pearson Correlation	0.601*	0.867*	0.618*	0.798*
	Sig. (2-tailed)	0.018	0.000	0.014	0.000

⁎ Correlation is significant at the 0.01 level (2-tailed).

The germination parameters (*GP, GI. MGT, MGR, CV_t_,* and *VI*) were calculated Eqs. 1 to (6), in addition to fresh weight (FW) and dry weight (DW) (see the germination parameters test file in the supplementary data). The calculated parameters of *Lens culinaris Medik* and *Vicia faba* seeds under the effect of bleach and vinegar treatments were displayed in Table 1.

Table 1 shows the effect of bleach and vinegar treatments on *GP, GI, MGT, MGR, CV_t_, VI*, in addition to *FW*, and *DW* in both seed types. The germination parameters were calculated using the equations from 1 to 6. Table 2 represents the correlation between *GP* on a side, and *GI, MGT, MGR, CV_t_*, and *VI* in lentil and faba bean, on the other side.

The variation in the lintel and faba bean seeds germination percentage (GP) between the treatments was displayed in Fig. 2.

The GP variation between treatments was measured using a one-way ANOVA test and Tukey's multiple comparison test (Table 3). Tukey Simultaneous GP test between treatments in both seed types at 95% Confidence interval *(CI)* was conducted referred to Fig 7 in the supplementary data, where (A) mean differences of GP in Lentil with bleach, (B) mean differences of GP in Lentil with vinegar, (C) mean differences of GP in faba bean with bleach, (D) mean differences of GP in faba bean with vinegar.

**Table 3:** One-way ANOVA test for Germination percentage (*GP*) coupled with germination index (*GI*) of *Lens Culinaris* and *Vicia faba* seeds treated with two commercial Sanitizers (bleach and vinegar).

			One-way ANOVA		
Crop seed	Sanitizers	Germination parameter	*SOV*	*DF*	*MS*
*Lens Culinaris*	Bleach	Germination percentage (GP)	Between Groups Within Groups	4 10	5426.667⁎⁎ 133.333
		Germination index (GI)	Between Groups Within Groups	4 10	15.644⁎⁎ 2.364
	Vinegar	Germination percentage (GP)	Between Groups Within Groups	4 10	8306.667⁎⁎ 106.667
		Germination index (GI)	Between Groups Within Groups	4 10	23.123⁎⁎ 1.180
*Vicia faba*	Bleach	Germination percentage (GP)	Between Groups Within Groups	4 10	3706.667⁎⁎ 266.667
		Germination index (GI)	Between Groups Within Groups	4 10	13.290⁎⁎ 1.049
	Vinegar	Germination percentage (GP)	Between Groups Within Groups	4 10	5133.333⁎⁎ 80.000
		Germination index (GI)	Between Groups Within Groups	4 10	15.218⁎⁎ 0.817

⁎⁎ Highly significant differences at a confidence interval of *P ≤ 0.01.*

The mean germination index (GI), mean germination time (MGT), and the mean germination rate (MGR) of Lentil with bleach treatments were illustrated in Fig. 3 referred to Fig 5 in the supplementary data.

Fig. 3 shows the (GI, MGT, and MGR) parameters in both seed types under bleach treatment with a significant variation at 0.5% and relatively at 0.1% concentrations, while the variation not evident in the lowest treatment levels (0.0%, 0.005%, and 0.05%). Table 3 represents the variations between Germination percentage (GP) coupled with the germination index (GI) as a representative evaluation of seed germination. The data referred to SPSS file in the supplementary data.

The vigour index has been calculated in both seeds under different treatment levels of bleach and vinegar because given the importance of the vigour index (VI) to measure the damage accumulations as viability decline [6]. The results of VI were shown as referred to Fig 6 in the supplementary data.

### 1.2 Instrumental analysis

The chloride (Cl^−^) and acetate [CH_3_COO]^−^ anions, in addition to phosphate, sulfate, sodium, ammonium, potassium, calcium, and magnesium, were analyzed by the ion chromatography (IC) method. The raw data for every independent analysis was represented in the supplementary data (see the chemical analysis files). Table 4 shows the chloride, sodium, and acetate ions concentrations were increased with increasing the treatment level. Other essential nutrients concentrations were reduced referred to the chemical analysis file and Fig 8 in the supplementary data.

**Table 4:** Per cent Anions and cations analysis in *Vicia faba* and *Lens culinaris* seedling using the ion chromatography method (*IC*) with 3 replicates.

Seeds	Treatment	Cl^−^	[C_2_H_3_O_2_]^−^	Na^+^	PO₄³⁻	SO_4_^2−^	NH_4_^+^	K^+^	Ca^2+^	Mg^2+^
*Vicia faba*	Control	0.08%	0.01%	0.09%	1.86%	0.49%	0.02%	3.38%	0.43%	0.66%
	Control	0.08%	0.01%	0.00%	1.89%	0.52%	0.04%	3.63%	0.43%	0.73%
	Control	0.08%	0.01%	0.00%	1.75%	0.51%	0.07%	3.45%	0.32%	0.66%
	0.005%	0.12%	0.66%	0.29%	1.54%	0.50%	0.00%	3.26%	0.25%	0.53%
	0.005%	0.15%	0.37%	0.19%	1.72%	0.63%	0.08%	3.76%	0.54%	0.56%
	0.005%	0.15%	0.71%	0.22%	1.73%	0.57%	0.04%	4.16%	0.24%	0.59%
	0.05%	0.19%	2.03%	0.63%	1.29%	0.37%	0.00%	2.59%	0.63%	0.61%
	0.05%	0.23%	1.14%	0.74%	1.49%	0.57%	0.04%	3.22%	0.54%	0.63%
	0.05%	0.23%	1.16%	0.73%	1.46%	0.49%	0.11%	3.52%	0.52%	0.60%
	0.1%	0.36%	1.33%	1.33%	1.32%	0.76%	0.06%	3.03%	0.46%	0.67%
	0.1%	0.29%	1.35%	1.13%	1.19%	0.60%	0.06%	2.61%	0.28%	0.50%
	0.1%	0.36%	1.16%	1.28%	1.23%	0.70%	0.04%	2.66%	0.64%	0.63%
	0.5%	2.75%	1.39%	3.47%	0.80%	0.40%	0.08%	1.03%	0.33%	0.45%
	0.5%	2.51%	2.32%	3.21%	0.77%	0.46%	0.00%	1.21%	0.23%	0.41%
	0.5%	2.46%	2.12%	3.13%	0.71%	0.42%	0.02%	0.95%	0.30%	0.42%

*Lens culinaris*	Control	0.03%	0.00%	0.03%	1.39%	0.55%	0.05%	2.33%	0.34%	0.41%
	Control	0.03%	0.00%	0.02%	1.43%	0.59%	0.06%	1.98%	0.00%	0.40%
	Control	0.02%	0.00%	0.02%	1.13%	0.47%	0.05%	1.61%	0.42%	0.51%
	0.005%	0.05%	0.00%	0.30%	1.31%	0.60%	0.00%	1.73%	0.32%	0.45%
	0.005%	0.05%	0.00%	0.32%	1.34%	0.64%	0.05%	1.85%	0.37%	0.46%
	0.005%	0.05%	0.00%	0.31%	1.40%	0.62%	0.07%	2.11%	0.35%	0.46%
	0.05%	0.20%	0.01%	1.01%	1.05%	0.48%	0.05%	0.45%	0.43%	0.48%
	0.05%	0.21%	0.01%	0.96%	0.93%	0.45%	0.06%	0.37%	0.28%	0.32%
	0.05%	0.18%	0.00%	0.95%	1.07%	0.47%	0.06%	0.38%	0.24%	0.36%
	0.1%	0.42%	0.28%	1.05%	0.79%	0.35%	0.00%	0.15%	0.10%	0.26%
	0.1%	0.35%	0.35%	1.01%	0.83%	0.35%	0.00%	0.20%	0.20%	0.27%
	0.1%	0.38%	0.24%	1.06%	0.91%	0.37%	0.16%	0.00%	0.06%	0.20%
	0.5%	2.64%	0.95%	2.60%	0.29%	0.05%	0.02%	0.05%	0.00%	0.08%
	0.5%	3.12%	1.15%	3.11%	0.33%	0.06%	0.00%	0.21%	0.00%	0.00%
	0.5%	2.47%	1.18%	2.57%	0.35%	0.06%	0.04%	0.20%	0.00%	0.03%

**Fig. 4:**
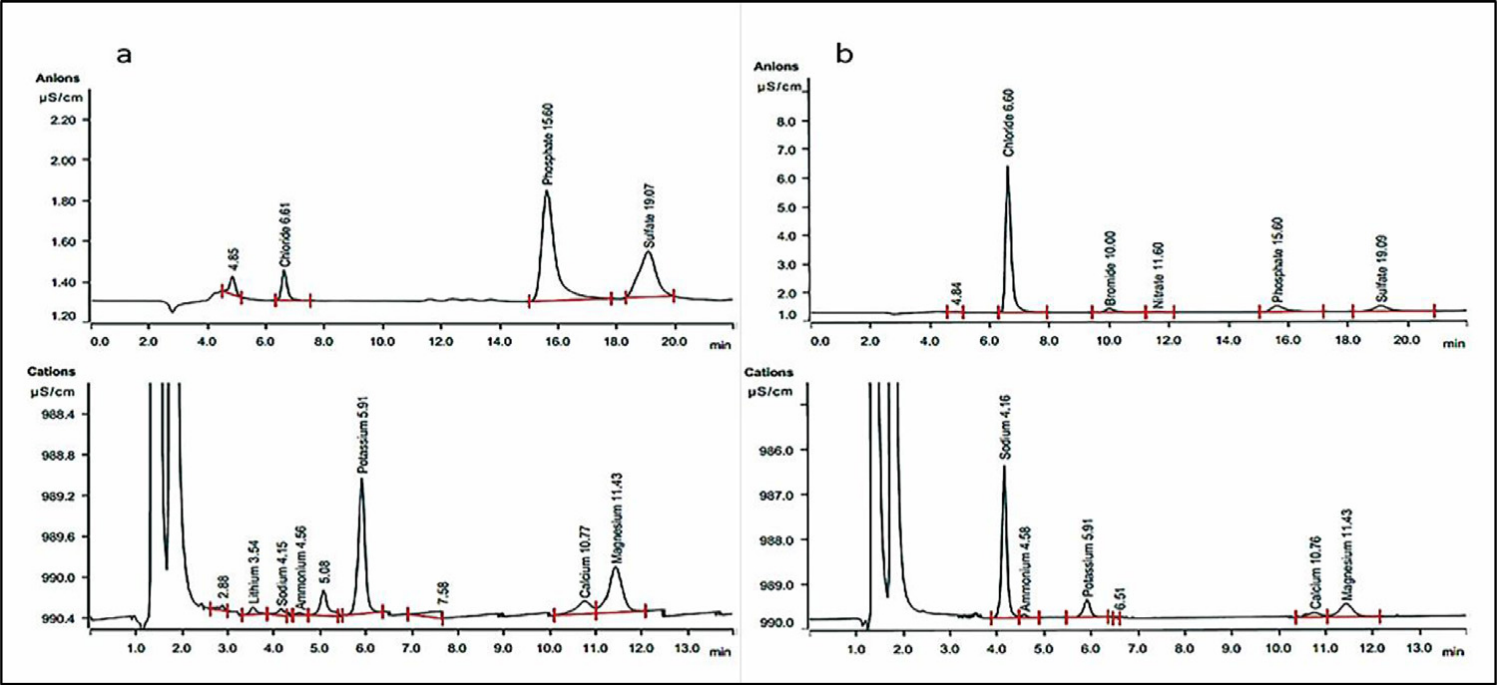
chromatogram of anions and cations that accumulated into seedling during the germination, (a) in the control sample, and (b) in the higher level of treatment (0.5%).

Fig. 4 demonstrates the chromatogram of the chemical ingredients (in the form of ions) accumulated from the source of bleach and vinegar during seven days of the seed's germination test.

## 2 Experimental Design, Materials and Methods

Two crop seeds (Lentil and Faba bean) were collected from Doha, Qatar's local market. Healthy seeds were selected and disinfected by soaking in bleach solution 10% for two minutes, followed by rinsing three times with sterilized distilled water before the treatment procedures. 5% Hypochlorite bleach (liquid bleach regular, Clorox, USA) and 5%white vinegar (Distilled white vinegar, Heinz, USA) treatment solutions were diluted to 0.5%, 0.1%, 0.05%, and 0.005% concentrations in addition to the control. The dilutions were carried on by dividing the initial concentration% multiplied by initial volume (mL) by the Final volume (mL). The final concentration% (active ingredients) is equivalent to the (v/v) concentration% (Table 5).

**Table 5:** Dilution Series of 5% hypochlorite bleach and 5% white vinegar treatment solutions.

Dilution	Initial volume (mL)	Initial Concentration%	Final volume (mL)	Final Concentration%	Final Concentration%(v/v)
1	10	5	100	0.5	10
2	2	5	100	0.1	2
3	1	5	100	0.05	1
4	0.1	5	100	0.005	0.1

Ten sterilized seeds were transferred into each Petri dish (NUNC Petri dishes diam. H 90 mm, Sigma-Aldrich, Germany) with a sterilized filter paper as an independent replicate. The Petri dishes were arranged according to the completely randomized design (CRD) with three replicates (see Fig 1 in the supplementary data). The germination test was carried out during seven days at the laboratory conditions; roots (radicle) were started growing on day one while shoots (plumule) were started on day three in both lentil and faba bean seeds. Seeds were counted as a germinated seed when it becomes > 2 mm long [8]. The maximum elongation (mm) of roots and shoots was recorded on the last day of the germination test under different treatments. A proper volume from each concentration was added (30 ml per dish), and a sterilized distilled water was added to the control dishes.

After seven days, the whole seedling samples were dried for 48h in a freeze dryer (AdVantage Pro series, SP Scientific-UK). Approximately 0.1g of the milled and homogenized sample was weighing in a 15ml centrifugal tube using an analytical balance (AS 220.R2 PLUS, RADWAG- Poland). Accurately, 3 ml of deionized water (18.2 MΩ cm-1 at 25 °C) was added to the sample for extraction and thin sonicated at 20 kHz for the next 60 seconds or until the plant tissue homogenized well using ultrasonic cleaner (Ultrasonic Cleaner 22L, VEVOR-USA). Tissue samples were centrifuged (SORVALL-ST16R, Thermo Fisher Scientific- US) at 5000 rpm for 15 min, and the supernatant was transferred to a fresh centrifuge tube (Chapp et al. 2018). The centrifugal process was repeated three times with 1ml of deionized water to maximize recovery%. The final extracted sample was diluted 100 times by adding 0.5 mL liquid sample to 49.5 ml of the reagent water in a 50 mL centrifuge tubes. The diluted samples were filtered through sterile PTFE syringe filters (0.2 μm) in a new, sterilized 15 mL centrifuge tubes. A liquate of samples were transferred to the ion chromatography vials and analyzed through the ion chromatography.

### 2.1 Chemical analysis

The standard solution of the acetate anions (1000 mg L^−1^) was prepared by weighing a proper mass of ammonium acetate (Laboratory reagent grade, ≥ 97%, Fisher Scientific-UK) and dissolving it in deionized water. A pure chloride standard (IC-MAN-02-1, AccuStandard, US) was used to prepare the chloride anions calibration curve. The acetate and chloride anions calibration curves were analyzed using Ion chromatography (882 Compact IC plus, Metrohm AG- Switzerland) with a dialysis system, and an isocratic pump used to perform the chemical analysis. NaHCO_3_ (99.7-100.3% ACS, VWR, USA) and Na_2_CO_3_ (anhydrous ≥ 99.5% ACS, VWR, USA) aqueous solution were used as the eluents in addition to H_3_PO_4_ as a regeneration solution [9]. IC column (Metrosep A Supp 5-150/4.0, Metrohm AG, Switzerland), (Metrosep C4-150/4.0, Metrohm AG, Switzerland) were used to separate anions and cations, respectively. An IC conductivity detector (850 Professional IC, Metrohm AG, Switzerland) was used for anions and cations analyses.

### 2.2 Quality assurance/quality control

Analytical quality assurance was established to check the performance of the sample preparation and analysis. The QC samples were constituted ten per cent of each batch consisted of reagent blank, samples triplicate, and spiked sample to achieve the QC criteria. The obtained recovery values showed averages of 87.6% to 96.8% in acetate and chloride, respectively.

### 2.3 Data analysis

The variation within different germination parameters was statistically tested under the effect of treatments using one-way ANOVA, Tukey's Studentized Range (HSD) for multiple comparisons with a significance level of *P* < 0.05, and Pearson correlations between treatments were examined using IBM-SPSS statistics version-25 and Minitab version-17.3.1.

## Ethics Statement

Not applicable.

## Declaration of Competing Interest

The authors declare that they have no known competing financial interests or personal relationships that could have appeared to influence the work reported in this paper.
